# From Forest to Fork: Antioxidant and Antimicrobial Potential of *Laetiporus sulphureus* (Bull.) Murrill in Cooked Sausages

**DOI:** 10.3390/microorganisms13081832

**Published:** 2025-08-06

**Authors:** Aleksandra Novaković, Maja Karaman, Branislav Šojić, Predrag Ikonić, Tatjana Peulić, Jelena Tomić, Mirjana Šipovac

**Affiliations:** 1 Faculty of Education, University of East Sarajevo, Semberskih Ratara E1, 76300 Bijeljina, Bosnia and Herzegovina; 2 Fungi Laboratory, Department of Biology and Ecology, Faculty of Sciences, University of Novi Sad, Trg Dositeja Obradovića 2, 21000 Novi Sad, Serbia; 3 Faculty of Technology Novi Sad, University of Novi Sad, Bulevar cara Lazara 1, 21000 Novi Sad, Serbia; 4 Institute of Food Technology in Novi Sad, University of Novi Sad, Bulevarcara Lazara 1, 21000 Novi Sad, Serbia; 5 Faculty of Technology, University of East Sarajevo Karakaj bb, 75400 Zvornik, Bosnia and Herzegovina

**Keywords:** edible mushrooms, natural preservatives, phenolic compounds, antioxidant activity, antimicrobial activity, *Laetiporus sulphureus*, cooked meat products, shelf life, food safety

## Abstract

In response to the growing demand for clean-label preservatives, this study investigates the potential of *Laetiporus sulphureus*, an edible polypore mushroom, as a multifunctional additive in cooked sausages. The ethanolic extract of *L. sulphureus* (LsEtOH) was evaluated for its chemical composition, antioxidant capacity, and antimicrobial activity. Leucine (12.4 ± 0.31 mg/g d.w.) and linoleic acid (68.6%) were identified as the dominant essential amino acid and fatty acid. LsEtOH exhibited strong antioxidant activity, with IC_50_ values of 215 ± 0.05 µg/mL (DPPH•), 182 ± 0.40 µg/mL (NO•), and 11.4 ± 0.01 µg/mL (OH•), and showed a selective inhibition of Gram-positive bacteria, particularly *Staphylococcus aureus* (MIC/MBC: 0.31/0.62 mg/mL). In cooked sausages treated with 0.05 mg/kg of LsEtOH, lipid peroxidation was reduced (TBARS: 0.26 mg MDA/kg compared to 0.36 mg MDA/kg in the control), microbial growth was suppressed (33.3 ± 15.2 CFU/g in the treated sample compared to 43.3 ± 5.7 CFU/g in the control group), and color and pH were stabilized over 30 days. A sensory evaluation revealed minor flavor deviations due to the extract’s inherent aroma. Encapsulation and consumer education are recommended to enhance acceptance. This is the first study to demonstrate the efficacy of *L. sulphureus* extract as a natural preservative in a meat matrix, supporting its application as a clean-label additive for shelf life and safety improvement.

## 1. Introduction

The growing consumer demand for healthier food products, combined with a heightened awareness of the potential risks associated with synthetic additives, has intensified the search for natural alternatives capable of preserving food quality and safety. Within this context, the concept of “clean label” has gained prominence, promoting the use of natural, functional, and sensory-acceptable ingredients [1]. The global clean-label ingredient market is projected to exceed USD 60 billion by 2027, reflecting a strong consumer preference for minimally processed and transparent food formulations [2].

Edible mushrooms, beyond their nutritional benefits, have emerged as promising yet underutilized sources of bioactive compounds with potent antioxidant and antimicrobial properties. They are appreciated for their high-quality proteins, balanced amino acid profiles, low fat content, and richness in dietary fiber and essential micronutrients. Moreover, they synthesize a broad range of primary and secondary metabolites—including polysaccharides, phenolic compounds, sterols, flavonoids, and terpenes—that contribute to their health-promoting potential [3,4,5].

Several studies have confirmed that mushroom-derived extracts can effectively improve the oxidative and microbiological stability of meat products, supporting their use as clean-label ingredients in food preservation [6,7,8]. This has further fueled interest in natural antimicrobials as alternatives to synthetic preservatives such as nitrites, particularly in minimally processed meat systems.

Among these species, *Laetiporus sulphureus* (commonly known as “chicken of the woods”) has attracted attention due to its distinctive flavor profile and its richness in phenolic compounds and fatty acids [9,10]. Although numerous studies have demonstrated the in vitro antioxidant and antimicrobial properties of its bioactive constituents, their efficacy in real food matrices, especially in meat products, remains underexplored [10]. Moreover, limited research has assessed the functional performance of mushroom extracts under realistic processing and storage conditions [11,12].

This study aimed to evaluate the application of an ethanolic extract of *L. sulphureus* (LsEtOH) as a natural preservative in cooked sausages. The extract was assessed for its ability to inhibit lipid oxidation, reduce microbial proliferation, and maintain key physicochemical and sensory attributes during 30 days of refrigerated storage. A fixed concentration was selected based on preliminary trials, and sensory testing was conducted to assess acceptability. To our knowledge, this is one of the first studies to investigate the multifunctional potential of *L. sulphureus* extract as a clean-label additive in a real meat system, combining chemical characterization with antioxidant, antimicrobial, and sensory evaluation. These findings may inform the development of sustainable preservation strategies and support the integration of wild mushroom extracts in functional meat products.

## 2. Materials and Methods

### 2.1. Chemicals and Reagents

Reference standards for phenolic compounds were obtained from Sigma-Aldrich (Steinheim, Germany), Fluka Chemie GmbH (Buchs, Switzerland), and Chromadex (Santa Ana, CA, USA). Methanol (HPLC grade) was purchased from J.T. Baker (Deventer, The Netherlands), and formic acid (p.a. grade) from Merck (Darmstadt, Germany). A standard FAME mixture (Supelco 37) was supplied by Sigma-Aldrich (Steinheim, Germany). Deionized water was produced using a Millipore purification system (Millipore, Darmstadt, Germany). All other solvents and reagents were of analytical grade and obtained from standard commercial suppliers.

### 2.2. Mushroom Collection and Sample Preparation

Wild fruiting bodies of the autochthonous *Laetiporus sulphureus* (Phylum Basidiomycota, Class Agaricomycetes, Order Polyporales, Family Laetiporaceae, Genus *Laetiporus*) were collected in the autumn season from Sikola, eastern Serbia. Species identification was confirmed by an experienced mycologist, and a voucher specimen (No. 12-00663) was deposited at the ProFungi Laboratory, Department of Biology and Ecology, Faculty of Sciences, University of Novi Sad, and the BUNS Herbarium. The harvested material was initially stored at −20 °C, then freeze-dried to a constant weight using a laboratory lyophilizer (Christ Alpha 1–2 LD; Martin Christ GmbH, Osterode am Harz, Germany). The dried biomass was ground to a fine powder, sealed in polyethylene bags, and kept at −20 °C until further analysis 10.

### 2.3. Ethanolic Extraction Procedure

A total of 10 grams of lyophilized *L. sulphureus* powder were extracted with 100 mL of 96% ethanol (EtOH) at room temperature (23 ± 1 °C) for 24 h on a rotary shaker (Thermo Fisher Scientific, Waltham, MA, USA) set at 120 rpm. The extraction conditions (solvent, time, and temperature) were selected based on previously published protocols [4], in order to ensure method reproducibility and to preserve thermolabile bioactive compounds. While further optimization may enhance compound recovery, the selected protocol offered a balanced compromise between efficiency and compound stability. The mixture was filtered through Whatman No. 4 filter paper (Whatman, GE Healthcare, Maidstone, UK), and the solvent was removed by rotary evaporation at 40 °C (Büchi, Flawil, Switzerland). The resulting dry extract was labeled as LsEtOH and stored at −20 °C until further analysis. All measurements were performed in triplicate.

### 2.4. Crude Protein Determination

Total protein content was determined by the macro-Kjeldahl method according to AOAC procedures (16th edition), using a nitrogen-to-protein conversion factor of 4.38 [13].

### 2.5. Protein Profiling

Protein profiling was conducted using a modified method based on Tidona et al. [14]. Briefly, approximately 15 mg of lyophilized *L. sulphureus* powder was suspended in 100 µL of extraction buffer (0.125 M Tris-HCl, 4% SDS, 2% glycerol, 2% β-mercaptoethanol; pH 6.8) and incubated at 100 °C for 5 min. Protein separation and analysis were performed with a chip-based Agilent 2100 Bioanalyzer (Agilent Technologies, Santa Clara, CA, USA) using the Protein 80 Plus Lab Chip kit (Agilent Technologies, Santa Clara, CA, USA), according to the manufacturer’s instructions. Bovine serum albumin (BSA) served as the molecular weight standard, and protein quantification was based on size distribution profiles [15]. All measurements were performed in triplicate.

### 2.6. Amino Acid Composition

Amino acid profiling was carried out after acid hydrolysis of the mushroom samples, following an established protocol [16]. The analysis was performed using high-performance liquid chromatography (HPLC) on an Agilent 1200 Series instrument (Agilent Technologies, Santa Clara, CA, USA) equipped with a fluorescence detector (excitation at 340 nm and emission at 450 nm) and an Agilent Eclipse Plus C18 column (5.0 μm, 3.0 × 250 mm). Concentrations were calculated using calibration curves derived from standard amino acid solutions (Sigma-Aldrich, Steinheim, Germany), and the final results are expressed in mg per g dry weight (mg/g d.w.). All measurements were performed in triplicate.

Concentrations were calculated using calibration curves derived from standard amino acid solutions, and the results are expressed in mg per g dry weight (mg/g d.w.).

### 2.7. Fatty Acid Analysis

Total lipid content was extracted using the Folch method [17], followed by transesterification with 14% boron trifluoride (BF_3_) in methanol [18]. The resulting fatty acid methyl esters (FAMEs) were analyzed using an Agilent 7890A gas chromatograph (Agilent Technologies, Santa Clara, CA, USA) equipped with a flame ionization detector (FID) and SP-2560 capillary column (100 m × 0.25 mm × 0.20 µm, Supelco, Sigma-Aldrich, Steinheim, Germany). Fatty acids were identified by comparison with a standard mixture of 37 FAMEs and quantified as percentages of total identified fatty acids.

### 2.8. Mineral Element Determination

Macroelements (K, Ca, Mg) and microelements (Fe, Zn, Mn, Cu) were quantified using flame atomic absorption spectrometry (AAS) following dry-ash digestion of the samples, as described in a previously validated method for mushroom matrices [16]. Mineral concentrations were expressed as mg/g dry weight (d.w.) for macroelements and mg/kg d.w. for microelements. All measurements were performed in triplicate.

### 2.9. Phenolic Profile Analysis

The qualitative and quantitative compositions of phenolic compounds in the ethanolic extract were analyzed using liquid chromatography–tandem mass spectrometry (LC–MS/MS), following the method described by Orčić et al. [19]. Compound identification was based on retention times and fragmentation patterns compared with authenticated standards. The results are expressed as micrograms per gram of dry extract (µg/g d.w.).

### 2.10. Total Phenolic Content (TPC) and Total Flavonoid Content (TFC)

Total phenolic content (TPC) was determined using the Folin–Ciocalteu reagent, following the method of Singleton et al. [20], adapted to a 96-well microplate format. Absorbance was measured at 765 nm using a Multiskan Ascent microplate reader (Thermo Electron Corporation, Waltham, MA, USA). Gallic acid was used to generate the standard calibration curve, and the results are expressed as milligrams of gallic acid equivalents (mg GAE) per 100 g of extract.

Total flavonoid content (TFC) was determined according to the aluminum chloride colorimetric method modified for microplate use [21]. Absorbance was read at 415 nm. Quercetin was used as the calibration standard, and the results are expressed as mg quercetin equivalents (QE) per 100 g of extract. All measurements were performed in triplicate

### 2.11. Antioxidant Activity Assays

The antioxidant capacity of the extract was assessed using three in vitro assays, DPPH•, NO•, and OH• radical scavenging activities, following the protocols described by Espin et al. [22], Green et al. [23], and Cheeseman et al. [24], respectively. Absorbance of the reaction mixtures was measured with a microplate reader. The radical scavenging capacity (RSC) was calculated using the following formula:

RSC (DPPH, NO, OH) (%) = (1 − A sample/A control) × 100%

The IC_50_ values (µg/mL), defined as the concentration required to inhibit 50% of radical activity, were calculated from dose–response curves. For this purpose, a series of LsEtOH concentrations ranging from 1.15 to 800 µg/mL was tested in all antioxidant assays (DPPH•, NO•, and OH•). Absorbance was measured spectrophotometrically, and solvent controls (5% DMSO without extract) were included to correct for background absorbance. The dried ethanolic extract (LsEtOH) was redissolved in 5% dimethyl sulfoxide (DMSO; Fluka Chimie) prior to analysis. All measurements were performed in triplicate.

### 2.12. Antibacterial Activity

Antibacterial effects were evaluated against five ATCC reference strains: *Staphylococcus aureus* ATCC 25923, *Bacillus subtilis* ATCC 6633, *Enterococcus faecalis* ATCC 19433, *Escherichia coli* ATCC 11229, and *Salmonella enteritidis* ATCC 13076. The broth microdilution method in 96-well plates was used to determine minimum inhibitory concentration (MIC) and minimum bactericidal concentration (MBC), following Karaman et al. [25]. The tested concentrations of LsEtOH ranged from 0.15 to 10 mg/mL. MIC was defined as the lowest concentration at which no visible bacterial growth was observed, confirmed by staining with TTC solution (2,3,5-triphenyltetrazolium chloride), which enhances the visualization of bacterial growth. MBC was determined by subculturing the contents of wells without visible growth onto nutrient agar. The extract was diluted in 5% DMSO; ampicillin and gentamicin served as positive controls.

### 2.13. Sausage Formulation and Processing

Cooked sausages were produced in a certified small-scale local meat processing facility using a standardized formulation consisting of chicken breast (25.0%), pork shoulder (16.7%), mechanically deboned chicken meat (16.7%), pork fat (16.7%), ice (16.7%), maize starch (3.3%), textured soy protein (2.2%), curing salt containing sodium nitrite (1.8%), spice blend (0.6%), polyphosphates (0.3%), and dextrose (0.1%). Due to food safety requirements and facility protocols, all sausage formulations included a minimal dose of curing salt (1.8%) to simulate realistic production conditions. All ingredients were homogenized using a bowl cutter (Taifun 200, Nowicki, Skwierzyna, Poland). The treatment batch was supplemented with *L. sulphureus* ethanolic extract (LsEtOH) at a final concentration of 0.05 mg/kg, while the control batch was prepared without the addition of the extract.

Both sausage batches were stuffed into 40 mm diameter polyamide casings and thermally processed in steam until a core temperature of 72 °C was achieved. Pasteurization was followed by rapid cooling using a water/air system to 25 °C. All samples were vacuum-packed and stored at 4 °C for 30 days. Three independent production batches were prepared for each treatment group.

### 2.14. pH and Color Measurement

The pH of the sausages was measured using a portable penetration-type pH meter (Testo SE & Co. KGaA, Lenzkirch, Germany). Measurements were performed in duplicate on three randomly selected sausages from each batch.

Color parameters—lightness (L), redness (a), and yellowness (b*)—were assessed on freshly cut surfaces using a CR-400 Chroma Meter (Konica Minolta, Tokyo, Japan) under D65 illumination. The instrument was calibrated with a standard white plate prior to measurement. Each value represents the mean of 15 readings obtained from three sausages per batch [26,27,28].

### 2.15. Lipid Oxidation Assay (TBARS)

Lipid oxidation was evaluated using the thiobarbituric acid reactive substances (TBARS) assay, following the method of Botsoglou et al. [29] with minor modifications [30]. The absorbance of the pink MDA–TBA complex was measured at 532 nm using a Jenway 6300 spectrophotometer (Cole-Parmer Ltd., Stone, Staffordshire, UK). The results are expressed as milligrams of malondialdehyde per kilogram of sausage sample (mg MDA/kg). Analyses were conducted in duplicate on three sausages per treatment group.

### 2.16. Microbiological Evaluation

Microbial load was evaluated by quantifying total aerobic mesophilic bacteria (AMB), *Escherichia coli*, *Clostridium* spp., *Enterobacteriaceae*, and yeasts/molds, following the method described by Šojić et al. [31]. The results are expressed as colony-forming units per gram (CFU/g). For samples marked as “not detected” (Nd), no colony growth was observed on the corresponding plates. The detection limit of the applied plating method was 10 CFU/g, corresponding to the lowest quantifiable count under the experimental conditions. Microbial counts were expressed as colony-forming units per gram (cfu/g). Analyses were conducted on three samples per batch on day 1 and day 30 of refrigerated storage.

### 2.17. Sensory Evaluation

Sensory attributes, specifically color and taste, were evaluated by a trained panel of seven assessors in accordance with international ISO standards [32,33,34]. Panelists were selected and trained following the guidelines of ISO 8586:2012, which define general principles for the selection, training, and monitoring of sensory assessors.

The training program included:

- Familiarization with key sensory characteristics relevant to cooked sausages (color and taste);
- Use of standardized terminology in accordance with ISO 5492:2008 to ensure consistency in the description and interpretation of sensory attributes;
- Application of structured quantitative sensory scales based on ISO 4121:2003, including calibration with reference samples to ensure reliable and repeatable scoring.

All evaluations were conducted in individual sensory booths under standardized environmental conditions [35]. Data were recorded using pre-designed scoring sheets. A six-point hedonic scale was used to assess overall sensory quality, where:

5 = excellent/typical, 4 = minor deviation, 3 = moderate defect, 2 = distinct defect, 1 = unacceptable, and 0 = spoilage/contamination.

Sensory evaluations were performed on days 1 and 30 of refrigerated storage in a controlled sensory analysis laboratory. The evaluation procedure, including the timing of assessments and sample handling, was based on previously published studies that assessed the effects of natural additives on the oxidative, microbial, and sensory stability of cooked sausages during chilled storage [31].

### 2.18. Statistical Analysis

All data are expressed as mean ± standard deviation (SD). Differences between control and treatment groups were analyzed using one-way ANOVA, followed by Duncan’s post hoc test for multiple comparisons. Statistical significance was set at *p* < 0.05. Analyses were performed using STATISTICA software (version 12.0, StatSoft, Inc., Tulsa, OK, USA).

## 3. Results and Discussion

### 3.1. Chemical Composition of L. sulphureus Extract

**Figure 1:**
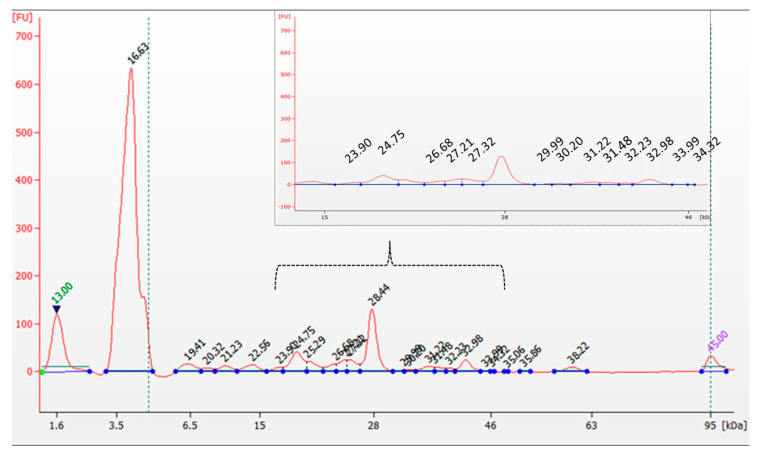
Electropherogram of analyzed *L. sulphureus*.

Mushroom-derived proteins are known for their favorable amino acid profiles, often containing all essential amino acids (EAAs) required for human nutrition [3,36] In our study, the protein content of *L. sulphureus* was determined to be 14.5%, with electrophoretic analysis identifying 24 distinct fractions (6.4–59.7 kDa), including a prominent band at 51.8 kDa (Figure 1). The amino acids most commonly detected in fungi include lysine, methionine, tryptophan, threonine, valine, leucine, isoleucine, histidine, and phenylalanine [3]. The amino acid composition of *L. sulphureus* determined in this study (Table 1) revealed particularly high concentrations of non-essential amino acids, such as cysteine (26.8 ± 0.20 mg/g d.w.), glutamic acid (26.2 ± 0.06 mg/g d.w.), arginine (23.3 ± 0.16 mg/g d.w.), and aspartic acid (16.1 ± 0.22 mg/g d.w.). Among the essential amino acids, leucine was the most abundant (12.4 ± 0.31 mg/g d.w.), followed by valine (9.80 ± 0.22 mg/g d.w.) and threonine (8.50 ± 0.17 mg/g d.w.). The total content of essential and non-essential amino acids was calculated as 53.98 mg/g and 131.51 mg/g d.w., respectively. These results are consistent with previous findings reported by Agafonova et al. for two *L. sulphureus* strains isolated from Siberia [37].

Beyond their nutritional role, several amino acids abundant in mushrooms, such as arginine, cysteine, and glutamic acid, are involved in immunomodulation, antioxidative defense, and neuroprotection, further enhancing the functional value of mushroom-derived proteins [38,39]. The presence of bioactive peptides and lectin-like proteins within mushroom matrices may also contribute not only to nutritional quality but to health promoting properties relevant for functional food applications [40,41].

The fatty acid composition was assessed via gas chromatography-mass spectrometry (GC-MS), identifying 15 individual fatty acids (Table 2). While mushrooms are generally characterized by their low lipid content (2.96–4.50 g/100 g d.w.) [11], they are appreciated for their high proportion of polyunsaturated fatty acids (PUFAs), known for promoting cardiovascular health [42].

In the sample studied, linoleic acid was predominant (68.6%), followed by oleic (11.0%) and palmitic acid (9.68%). PUFAs accounted for 70.54% of the total fatty acids, while saturated fatty acids (SFAs) and monounsaturated fatty acids (MUFAs) constituted 16.83% and 12.62%, respectively. Compared to the previously reported data for *L. sulphureus* from Serbia [10], this profile suggests a favorable fatty acid composition, likely influenced by the local habitat environmental impacts.

LsEtOH exhibited a total phenolic content of 78.1 ± 0.40 mg GAE/100 g (d.w.) and a flavonoid content of 6.40 ± 0.10 mg QE/100 g d.w. LC–MS/MS analysis identified *p*-coumaric acid at 0.30 ± 0.01 µg/g d.w., suggesting its contribution to the extract’s antioxidant activity. This mycochemical profile underscores the species’ potential for developing nutraceuticals targeting oxidative stress management and metabolic health support [10].

Mineral analysis revealed potassium (K) as the predominant microelement (21.5 ± 0.30 mg/g d.w.), followed by magnesium (Mg) and calcium (Ca) (Table 3). Among microelements, zinc (Zn) was most abundant (58.3 ± 0.50 mg/kg d.w.), followed by copper (Cu), manganese (Mn), and iron (Fe). These findings align with previous reports that highlight potassium as the major microelement in mushrooms [43,44]. Elevated K and Zn levels may reflect adaptive mineral uptake related to environmental factors [3].

### 3.2. In Vitro Antioxidant and Antimicrobial Activities of L. sulphureus Extract

The ethanolic extract of *L. sulphureus* (LsEtOH) exhibited pronounced antioxidant activity, with IC_50_ values of 215 ± 0.05 µg/mL for DPPH•, 182 ± 0.40 µg/mL for NO•, and 11.4 ± 0.01 µg/mL for OH• radicals (Table 4). These results confirm the extract’s ability to effectively neutralize both oxygen and nitrogenderived reactive species.

In comparison to previous studies, the LsEtOH extract demonstrated a stronger OH• scavenging capacity than the methanolic extract reported by Karaman et al., which achieved 57.06% inhibition at a concentration of 400 µg/mL [45]. Conversely, the ethanolic extract of *Meripilus giganteus* showed even greater efficacy, with an IC_50_ of 1.74 ± 0.2 µg/mL for OH• and an IC_25_ of 148.04 ± 4.98 µg/mL for NO• radicals [46]. The NO• scavenging effect of our LsEtOH extract (IC_50_ = 182 ± 0.40 µg/mL) was comparable, indicating its relevance in modulating nitrogen-based radical species.

Regarding the DPPH• assay, the LsEtOH extract exhibited superior activity compared to polysaccharide-rich fractions of *L. sulphureus* studied by Klaus et al. Their most active alkaline-extracted fraction displayed an EC_50_ value of 500 ± 200 µg/mL [47], more than twice the concentration required to achieve a similar effect in our ethanolic extract.

These comparisons highlight the broad spectrum and potent radical scavenging potential of the LsEtOH extract, particularly its high efficacy against hydroxyl radicals.

The antioxidant potential of *L. sulphureus* extract can be primarily attributed to its rich phenolic profile, notably including compounds such as *p*-coumaric acid. Phenolic constituents are well known for their capacity to donate hydrogen atoms or electrons, thereby neutralizing free radicals and preventing oxidative damage in both biological and food matrices [48]. The activity of *p*-coumaric acid has been validated in situ, where it effectively scavenged reactive oxygen and nitrogen species and contributed to oxidative stability in food systems [49]. Its functional role has also been linked to dual mechanisms of microbial and oxidative inhibition [50], further supporting its contribution to the multifunctional properties of the extract.

In addition, the presence of polyunsaturated fatty acids, particularly linoleic and oleic acids, may play a synergistic role in enhancing antioxidant defenses. These fatty acids can contribute to membrane stabilization and reduce lipid peroxidation, thereby complementing the radical scavenging effects of phenolic compounds [51].

The ethanolic extract of *Laetiporus sulphureus* (LsEtOH) demonstrated selective inhibitory activity against Gram-positive bacteria (Table 5), with the strongest effect on *Staphylococcus aureus* (MIC/MBC: 0.31/0.62 mg/mL). This level of activity is comparable to that of thyme and oregano essential oils, which typically exhibit MIC values ranging from 0.25 to 0.5 mg/mL against *S. aureus* [52,53].

Moderate inhibition was observed against *Enterococcus faecalis* (2.50/10.0 mg/mL), which is in line with previously reported MIC values for rosemary and sage extracts (2–8 mg/mL) [54]. In contrast, the extract showed weak activity against *Bacillus subtilis* (10.0/10.0 mg/mL), whereas most plant-based extracts report MIC values below 2 mg/mL for this strain [54].

Compared to widely used plant-derived antimicrobials, LsEtOH exhibits promising efficacy against *S. aureus*, a major foodborne pathogen. This activity is likely due to the synergistic effects of phenolic acids and fatty acids present in the extract [49,51].

These findings support the potential application of *Laetiporus sulphureus* as a natural preservative, particularly in formulations targeting Gram-positive spoilage organisms. The selective activity of the LsEtOH extract aligns with previous observations that Gram-negative bacteria are generally more resistant to phenolic compounds. This resistance is largely attributed to the structural properties of their outer membrane, which limits permeability and restricts access of antimicrobial agents to intracellular targets [55].

Phenolic acids, such as *p*-coumaric acid, are known to exert a dual antimicrobial mode of action. Recent studies have shown that *p*-coumaric acid compromises bacterial cell membrane integrity, leading to leakage of intracellular contents and membrane hyperpolarization. Additionally, it interacts with genomic DNA, thereby interfering with replication and transcription processes [55].

Despite these potent mechanisms, the efficacy of *p*-coumaric acid and related phenolics is notably reduced against Gram-negative bacteria. This is due to the combined effect of their protective outer membrane, active efflux pumps, and enzymatic detoxification systems, all of which contribute to their diminished susceptibility to natural antimicrobials [56,57,58].

These findings support the potential application of *L. sulphureus* extracts in the development of multifunctional natural preservatives or biobased formulations targeting oxidative and microbial degradation in food and agricultural systems. Studies have demonstrated the species’ rich composition of phenolic compounds and its efficacy in prolonging shelf life [9,10], while broader investigations into wild edible mushrooms also support the role of fungal metabolites in food preservation and functional applications [59].

While the extraction parameters in this study were adapted from previous protocols [4], there is still room for further optimization. Future research could benefit from the application of multifactorial experimental designs, such as Response Surface Methodology (RSM), to systematically explore the influence of extraction variables on both yield and bioactivity. Building on the current methodological framework, such approaches may lead to more efficient and targeted extraction strategies in future studies.

### 3.3. Preserving Properties of Sausages

This section presents the influence of *L. sulphureus* ethanolic extract (LsEtOH) on key physicochemical, oxidative, microbiological, and sensory parameters of cooked sausages during 30 days of refrigerated storage. The results offer an integrative perspective on its preservative capacity.

Physicochemical Parameters: Initial pH values were comparable between the control (6.24 ± 0.1) and LsEtOH-treated sausages (6.23 ± 0.1). After 30 days of storage, a slight increase was recorded in both groups (6.26 ± 0.1 in control and 6.28 ± 0.1 in treated), with no statistically significant differences (*p* > 0.05) (Table 6). This suggests minimal proteolysis and stability of the protein matrix in the presence of fungal extract. Regarding color attributes (CIE L*, a*, b*), a marginal reduction in lightness and increase in redness was observed in treated samples. These changes are likely attributed to pigment myoglobin interactions or to direct coloration from *L. sulphureus*’s naturally vivid pigments, which may act as natural coloring agents [60]. Though subtle, such modifications could be advantageous in formulations targeting clean label status or seeking naturally enhanced visual appeal.

Lipid Oxidation: TBARS values increased in both groups over the storage period; however, sausages supplemented with LsEtOH showed significantly lower TBARS levels (0.26 ± 0.02 mg MDA/kg) compared to the control group (0.36 ± 0.02 mg MDA/kg) by day 30 (*p* < 0.05) (Table 7). Notably, all values remained below the sensory threshold of 1.0 mg MDA/kg, above which rancidity becomes organoleptically detectable [61]. These results highlight the antioxidant capacity of the extract, attributed mainly to phenolic acids, such as *p*-coumaric acid and membrane stabilizing polyunsaturated fatty acids. Similar antioxidant effects have also been reported for extracts derived from other medicinal mushrooms [49,51].

Microbiological Stability: During 30 days of refrigerated storage, sausages treated with LsEtOH consistently exhibited lower total aerobic mesophilic bacterial (AMB) counts compared to the untreated control. On day 1, bacterial counts reached 26.6 ± 11.5 cfu/g in the control samples, whereas treated sausages showed significantly lower counts of 16.6 ± 5.7 cfu/g. This difference persisted through day 30, with 43.3 ± 5.7 cfu/g in control versus 33.3 ± 15.2 cfu/g in the LsEtOH group (*p* < 0.05) (Table 8). Pathogens, such as *Escherichia coli*, *Clostridium* spp., *Salmonella enterica*, and molds were absent in all samples, likely reflecting the effectiveness of thermal processing and stringent hygienic production practices. The antimicrobial activity of the fungal extract is likely attributable to its secondary metabolites, particularly phenolic compounds and fatty acids, which are known to disrupt microbial membrane integrity and metabolic functions [10,11,12].

These findings support the potential application of *L. sulphureus* extracts in the development of multifunctional natural preservatives or bio-based formulations targeting oxidative and microbial degradation in food systems. Previous studies confirmed the rich phenolic profile and antioxidant activity of *L. sulphureus* primarily via in vitro analyses [10,11,12]. However, to the best of our knowledge, this is the first study to evaluate the preservative efficacy of *L. sulphureus* extract in a real meat matrix specifically, cooked sausages.

In comparison, *Ganoderma lucidum* extract has been added to pork products at 200–1000 mg/kg to enhance oxidative stability and reduce heterocyclic amines, and formulations using *Pleurotus ostreatus* puree (10–40%) in pork sausages achieved 24–37% lipid oxidation reduction over 15 days without sensory drawbacks. Additionally, *Agaricus brasiliensis* extract (0.5–1%) demonstrated antioxidant and antimicrobial results in pork patties like BHT (0.02%) over 9 days of chilled storage [62,63,64,65].

Based on the MIC determined for *S. aureus,* a sub-inhibitory concentration of 0.05 mg/kg was selected for incorporation into cooked sausages. This approach aimed to ensure sufficient antimicrobial and antioxidant functionality while avoiding potential sensory drawbacks associated with higher extract levels. Although the applied dose was lower than the typical concentrations used for plant-derived preservatives (e.g., 500–2000 mg/kg for thyme or rosemary extracts), it yielded measurable effects and aligns with clean-label formulation goals. Similar low-dose efficacy has been observed with essential oils, such as nutmeg (*Myristica fragrans*), which showed significant preservative potential in cooked sausages at only 10–20 ppm [31].

The novelty and practical relevance of our work positions *L. sulphureus* extract as a promising clean-label preservative with low-dose efficacy and multifunctional bioac-tivity.

**Figure 2:**
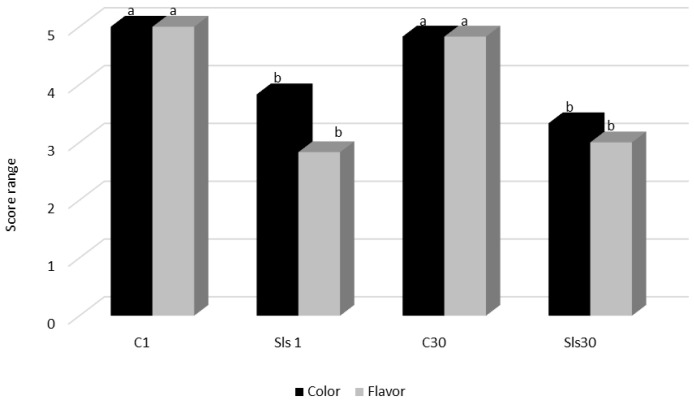
Effect of LsEtOH on the flavor and color of cooked sausages during storage. Color and flavor scores (mean ± SD) of cooked sausage samples evaluated by a sensory panel. Different letters (a, b) above the bars indicate statistically significant differences (*p* < 0.05) between samples within the same sensory attribute.

Sensory Evaluation: A trained sensory panel comprising seven individuals evaluated the organoleptic properties of the sausage samples. Sausages formulated with 0.05 mg/kg LsEtOH received significantly lower flavor acceptability scores compared to the control group (*p* < 0.05), likely attributable to the extract’s inherent taste and aroma. Sensory evaluations indicated noticeable differences in flavor and aroma, while color remained within acceptable sensory thresholds (Figure 2). To mitigate these flavor-related drawbacks, future product development could explore advanced formulation strategies, such as microencapsulation or nanoemulsion systems, which allow the controlled release of bioactive compounds and can effectively mask off-notes while preserving antimicrobial and antioxidant efficacy [66].

The concentration of 0.05 mg/kg was selected based on preliminary internal tests that indicated good product stability during refrigerated storage. Although a sensory evaluation was not conducted at that stage, the results support its use in this study. The low dose was chosen to avoid sensory alteration, commonly seen with higher concentrations of natural extracts. Despite its low level, this dose produced measurable antioxidant and antimicrobial effects and is markedly lower than typical concentrations of plant-based preservatives (500–2000 mg/kg) or synthetic additives, like sodium nitrite (100–150 mg/kg) [31].

Importantly, the distinctive aroma noted in LsEtOH-treated sausages reflects a well-recognized sensory compromise frequently observed in functional food products. Previous studies indicate that consumer receptivity to novel flavor profiles may improve when the health benefits are clearly communicated. One comprehensive review highlighted perceived health value as a key determinant of consumer acceptance of functional foods [67]. Accordingly, strategic marketing and consumer education focused on the scientifically supported antioxidant and antimicrobial benefits of *L. sulphureus* may enhance market acceptance and shift perception toward its unique sensory attributes.

## 4. Conclusions

This study provides a comprehensive characterization of *Laetiporus sulphureus* collected from eastern Serbia, emphasizing its potential as a multifunctional, clean-label ingredient for meat preservation. The ethanolic extract (LsEtOH) exhibited notable antioxidant and antimicrobial activity in cooked sausages, effectively reducing lipid oxidation and microbial proliferation over 30 days of refrigerated storage. These findings support the practical application of wild edible fungi-derived extracts in functional food systems. This study’s limitations include the use of a single extract concentration, limited sampling intervals, and a sensory analysis focused solely on flavor. Nevertheless, this work represents the first report to validate the preservative efficacy of *L. sulphureus* in a real meat matrix, offering novel insights beyond previous in vitro studies. Future research should focus on dose optimization, extended sensory profiling (including texture and appearance), time-resolved stability studies, and the isolation of individual bioactive compounds. Overall, LsEtOH emerges as a promising candidate for natural food preservation, aligning with current consumer trends for safety, sustainability, and transparency.

## Figures and Tables

**Table 1:** Amino acid composition of *L. sulphureus* (mg/g d.w.).

Amino Acid	Content (mg/g d.w.)	Classification
Valine (Val)	9.80 ± 0.22	Essential
Methionine (Met)	1.50 ± 0.10	Essential
Phenylalanine (Phe)	5.40 ± 0.07	Essential
Isoleucine (Ile)	5.90 ± 0.11	Essential
Leucine (Leu)	12.4 ± 0.31	Essential
Lysine (Lys)	7.60 ± 0.13	Essential
Threonine (Thr)	8.50 ± 0.17	Essential
Histidine (His)	2.90 ± 0.05	Essential
Aspartic acid (Asp)	16.1 ± 0.22	Non-Essential
Glutamic acid (Glu)	26.2 ± 0.06	Non-Essential
Serine (Ser)	9.30 ± 0.27	Non-Essential
Glycine (Gly)	8.00 ± 0.12	Non-Essential
Proline (Pro)	4.40 ± 0.09	Non-Essential
Arginine (Arg)	23.3 ± 0.16	Non-Essential
Alanine (Ala)	14.4 ± 0.15	Non-Essential
Tyrosine (Tyr)	3.00 ± 0.07	Non-Essential
Cysteine (Cys)	26.8 ± 0.20	Non-Essential

**Table 2:** The fatty acid composition of *L. sulphureus* relative %.

Fatty Acid Carbon Numbers	Common Name	%
C6:00	Caproic	1.24
C8:00	Caprylic	0.60
C14:00	Myristic	1.03
C15:00	Pentadecanoic	1.58
C16:00	Palmitic	9.68
C17:00	Heptadecanoic	0.68
C17:01	Heptadecanoic (cis-10)	0.31
C18:00	Stearic	1.61
C18:1n9c	Oleic	11.0
C18:2n6c	Linoleic	68.6
C18:3n6	Linolenic	0.83
C20:3n3	Eicosatrienoic	0.44
C20:4n6	Arachidonic	0.45
C22:1n9	Erucic	1.31
C23:00	Tricosylic	0.42

**Table 3:** Mineral composition of *L. sulphureus*.

Macroelement	mg/g d.w.
K	21.5± 0.30
Mg	0.84 ± 0.02
Ca	0.62 ± 0.01
Microelement	mg/kg d.w.
Cu	7.00 ± 0.10
Zn	58.3 ± 0.50
Mn	2.11 ± 0.10
Fe	0.09 ± 0.01

Values are means of three determinations ± standard deviation.

**Table 4:** Total phenolic/flavonoid content and IC50 (µg/mL) for radical scavenging DPPH•, NO• and •OH activity.

TPC Total Polyphenols (mg GAE/100 g d.w.)	TFC Total Flavonoid (mg QE/100 g d.w.)	DPPH•, IC50 (µg/mL)	NO•, IC50 (µg/mL)	•OH IC50 (µg/mL)
78.1 ± 0.40	6.4 ± 0.10	215 ± 0.05	182 ± 0.40	11.4 ± 0.01

Values are means of three determinations ± standard deviation.

**Table 5:** MIC and MBC (mg/mL) of LsEtOH tested strains of Gram-positive and Gram-negative bacteria.

	*S. aureus* ATCC25923	*E. faecalis* ATCC 19433	*B. subtilis* ATCC6633	*E. coli* ATCC11229	*S. enteritidis* ATCC 13076
MIC	0.31 mg/mL	2.50 mg/mL	10.0 mg/mL	>10.0 mg/mL	>10.0 mg/mL
MBC	0.62 mg/mL	10.0 mg/mL	10.0 mg/mL	>10.0 mg/mL	>10.0 mg/mL

The determination of MIC and MIC was performed in triplicate.

**Table 6:** Effect of LsEtOH on pH and color (CIE L*a*b*) of cooked sausages (SLs) during storage.

Storage Time (Days)	Batch	pH	CIE L * Value	CIE a * Value	CIE b * Value
1	C	6.24 ± 0.1 ^a^	69.3 ± 0.59 ^a^	15.9 ± 0.28 ^a^	16.3 ± 0.48 ^b^
	SLs	6.23 ± 0.1 ^a^	69.2 ± 0.57 ^a^	16.5 ± 0.19 ^ab^	15.1 ± 0.57 ^a^
30	C	6.26 ± 0.1 ^b^	69.1 ± 0.67 ^a^	16.6 ± 0.34 ^ab^	16.0 ± 0.15 ^b^
	SLs	6.28 ± 0.1 ^b^	68.7 ± 0.72 ^a^	16.9 ± 0.35 ^b^	14.7 ± 0.46 ^a^

^a,b^ Mean ± SD with different superscript letters in the same column (corresponding to the same phase of storage) differ significantly (*p* < 0.05). Batch C: without LsEtOH; SLs: with addition of LsEtOH. Values are means of three determinations ± standard deviation.

**Table 7:** Effect of LsEtOH on TBARS values (mg MDA/kg) of SLs during storage.

Storage Time (Days)	Batch	TBARS mg MDA/kg
1st	C	0.25 ± 0.02 ^ab^
	LsS	0.21 ± 0.02 ^a^
30th	C	0.36 ± 0.02 ^b^
	LsS	0.26 ± 0.02 ^ab^

^a,b^ Mean ± SD with different superscript letters in the same column (corresponding to the same phase of storage) differ significantly (*p* < 0.05).

**Table 8:** Effect of LsEtOH on microbiological profile (cfu/g) of cooked sausages during storage.

Microorganism	Storage Day	Batch C (CFU/g)	Batch SLs (CFU/g)
Aerobic mesophilic bacteria	1	26.6 ± 11.5 ᵃ	16.6 ± 5.7 ᵃ
	30	43.3 ± 5.7 ᵇ	33.3 ± 15.2 ᵃ
Yeasts and molds	1	Nd	Nd
	30	Nd	Nd
*Escherichia coli*	1	Nd	Nd
	30	Nd	Nd
*Clostridium* spp.	1	Nd	Nd
	30	Nd	Nd
*Enterobacteriaceae*	1	Nd	Nd
	30	Nd	Nd

^a,b^ Mean ± SD with different superscript letters in the same column (corresponding to the same phase of storage) differ significantly (*p* < 0.05), Nd—not detected; no colony growth observed on plates.

## Data Availability

All data supporting the findings of this study are contained within the article.
